# Granulocytic myeloid-derived suppressor cells expand in cord blood and human pregnancy and modulate T cell responses

**DOI:** 10.1186/2194-7791-1-S1-A14

**Published:** 2014-09-11

**Authors:** Natascha Köstlin, Hellen Kugel, Nikolaus Rieber, Bärbel Spring, Alexander Marmé, Christian F Poets, Christian Gille

**Affiliations:** Tübingen University Children’s Hospital, Department of Neonatology, Tübingen, Germany; Tübingen University Childrens Hospital, Department of Pediatrics I, Tübingen, Germany; Gynecology Practice, Am Lustnauer Tor 4, 72074 Tübingen, Germany

## Background

Preterm delivery is a leading cause of perinatal morbidity. Reasons are diverse, but immunologic fetal rejection has repeatedly been postulated to be involved. Myeloid derived suppressor cells (MDSC) are myeloid progenitor cells, characterized by their T cell suppressive capacity. They have predominantly been described under pathological conditions like cancer or infectious diseases, yet their role for materno-fetal tolerance remains elusive. We aimed to quantify and characterize MDSC in cord blood (CB) and in pregnancy.

## Methods

Cord blood mononuclear cells (CBMC) and peripheral blood mononuclear cells (PBMC) were prepared from CB of healthy newborns and blood of healthy pregnant women. Proportions of granulocytic MDSC (GR-MDSC, CD66b^+^/CD33^+^/CD14^-^/HLA-DR^+^ cells) and monocytic MDSC (MO-MDSC, CD14^+^/HLA-DR^-^ cells) were quantified, expression of enzymes arginase I (ArgI) and inducible NO-Synthetase (iNOS) was analysed by flow cytometry and production of reactive oxygen species (ROS) tested. Suppressive capacity of GR-MDSC on T cells was analysed in CFSE proliferation assay.

## Results

Percentages of GR-MDSC were up to tenfold higher in CBMC and in PBMC from pregnant women in all three trimesters compared to non-pregnant controls, whereas proportions of MO-MDSC did not change. GR-MDSC from CB and pregnant women expressed the enzymes ArgI and iNOS and produced high amounts of ROS. Addition of CB-GR-MDSC or GR-MDSC from pregnant individuals to PBMC from non-pregnant controls reduced the CD4 and CD8 T cell proliferation rate significantly compared to T cell proliferation alone.

## Conclusion

We describe for the first time an expansion of GR-MDSC in CB and during normal human pregnancy. Increased MDSC activity could play a role for induction and maintenance of materno-fetal immune tolerance and serve as a target for the therapy of immunologic pregnancy complications thereby preventing premature delivery.

